# Ultrasonography versus magnetic resonance imaging in detecting and grading common extensor tendon tear in chronic lateral epicondylitis

**DOI:** 10.1371/journal.pone.0181828

**Published:** 2017-07-27

**Authors:** Artur Bachta, Krzysztof Rowicki, Bartłomiej Kisiel, Magdalena Żabicka, Sylwia Elert-Kopeć, Janusz Płomiński, Witold Tłustochowicz, Artur Maliborski

**Affiliations:** 1 Department of Internal Diseases and Rheumatology, Military Institute of Medicine, ul. Szaserów 128, Warszawa, Poland; 2 Musculoskeletal Ultrasound Office, Military Institute of Medicine, ul. Szaserów 128, Warszawa, Poland; 3 Department of Orthopedics, Military Institute of Medicine, ul. Szaserów 128, Warszawa, Poland; 4 Department of Radiology, Military Institute of Medicine, ul. Szaserów 128, Warszawa, Poland; Universidad de Navarra, SPAIN

## Abstract

**Objectives:**

To investigate the diagnostic performance and reliability of ultrasonography (US) in detecting and grading common extensor tendon (CET) tear in patients with chronic lateral epicondylitis (LE), using magnetic resonance imaging (MRI) as the reference standard.

**Materials and methods:**

The study comprised fifty-eight chronic LE patients. Each patient underwent US and MRI. CET status was classified as: high-grade tear (≥50% thickness), low-grade tear (<50% thickness), suspected tear (possible but not evident tear), no tear. Additionally, the following dichotomous scale was used: confirmed or unconfirmed CET tear. Relative US parameters (versus MRI) for detecting CET tear included: sensitivity, specificity, positive predictive value (PPV), negative predictive value (NPV) and accuracy. The agreement between US and MRI findings was measured using the weighted Cohen kappa coefficient (κ).

**Results:**

US showed moderate agreement with MRI in detecting and grading CET tear (κ = 0.49). Sensitivity, specificity, and accuracy in CET tear detecting by US were 64.52%, 85.19%, and 72.73%, respectively. PPV and NPV of US were 83.33% and 67.65%, respectively. No patient with unconfirmed CET tear on US had high-grade CET tear on MRI.

**Conclusion:**

Ultrasonography is a valuable imaging modality that can be used as a screening tool to exclude high-grade CET tear in chronic LE patients. Once a tear is evident on US, MRI should be considered to assess precisely the extent of tendon injury.

## Introduction

Tendinopathy of the common extensor tendon (CET) at the lateral humeral epicondyle, also called lateral epicondylitis (LE), tendinosis of CET, lateral epicondylalgia or tennis elbow, affects up to 1.3% of the general population [1]. The disease is usually caused by a pathology of CET enthesis, mainly the enthesis of extensor carpi radialis brevis tendon. LE is characterized by pain and tenderness over the lateral humeral epicondyle and pain on resisted dorsiflexion and radial deviation of the wrist. LE is usually diagnosed clinically and diagnostic imaging is reserved for the cases resistant to conventional treatment.

Non-operative treatment, including activity modification, non-steroidal anti-inflammatory drugs, physiotherapy, corticosteroid injections, whole blood and platelet rich plasma injections, microtenotomy and shock-wave therapy, results in successful resolution of symptoms in 90% of patients [2–4]. An indication for surgical intervention is usually given after conservative treatment for 3–6 months has failed [5]. However, the study by Clarke et al. [6] showed that patients with a large intrasubstance tear or tears are less likely to respond to non-operative treatment, which suggests that a threshold for surgery may be lower in those patients. Thus, identifying patients with LE complicated by large CET tear, seems to be important in predicting disease outcome.

Currently, magnetic resonance imaging (MRI) is widely accepted as the most reliable imaging modality for diagnosing chronic elbow pain. However, the widespread use of MRI is limited by its high cost and several contraindications for its use. Ultrasonography (US) offers several advantages over MRI: it is easily accessible, non-invasive, cost-effective, and lacks any contraindications. Thus, US is considered as an important tool in the diagnosis of tendon problems, including LE.

Currently, studies evaluating the ability of US to detect CET tear, in comparison with MRI, are lacking. The purpose of this study was to investigate the diagnostic performance and reliability of US in detecting and grading CET tear, using MRI as the reference standard.

## Material and methods

### Study population

Between November 2013 and February 2015 we prospectively included 58 consecutive chronic LE patients. LE diagnosis was based on typical clinical symptoms and signs. Inclusion criteria was chronic LE (duration ≥3 months) resistant to conservative treatment. Exclusion criteria included the history of elbow injury or elbow surgery, the presence of symptoms or signs that may indicate synovitis or cervical radiculopathy. The diagram that shows flow of participants through the study is presented in S1 File. The study was approved by the Ethics Committee of Military Institute of Medicine. Each participant signed an informed consent form. All procedures were performed in accordance with the Helsinki Declaration of 1975, as revised in 1983.

### Ultrasonography

All ultrasound examinations were performed on the same Esaote MyLab ClassC (Esaote CA, USA) with a 6–18 MHz linear array transducer. All patients were examined by the same ultrasonographer (A.B.) with 15 years of experience in musculoskeletal ultrasonography. The observer was blinded to clinical details and MRI findings. CET status was classified as: high-grade tear (involving ≥50% of the CET thickness), low-grade tear (involving <50% of the CET thickness), suspected CET tear (possible but not evident CET tear), no CET tear. Additionally, the following dichotomous scale was used: confirmed CET tear (high-grade or low-grade CET tear) or unconfirmed CET tear (no CET tear or suspected CET tear).

### Magnetic resonance imaging

MRI was performed with a 3.0 T scanner (Discovery 750w, GE Medical Systems) using an 8 channel HD TR knee array coil or a 16 channel GEM flex coil. The scanning protocol included: axial T1-weighted spin-echo images [field of view (FOV) 120–140 x 120–140 mm, matrix 320 x 192, number of excitations (NEX) 2.0, slice thickness (ST) 2 mm], coronal T1-weighted fast spin-echo images (FOV 160 x 160 mm, matrix 320 x 224, NEX 2.0, ST 2 mm), sagittal and coronal T2-weighted fast spin-echo images with fat saturation (FOV 160 x 160 mm, matrix 320 x 224, NEX 2.0, ST 2 mm), axial PD-weighted images with fat saturation (FOV 120–140 x 120–140 mm, matrix 288 x 288, NEX 2.0, ST 2 mm) and after intravenous injection of standard dose 0.1 ml/kg of a paramagnetic contrast agent. However, it should be emphasized that only non-contrast MR images were used for the assessment of CET tendon injury (contrast MR images were performed for the purpose of future evaluation, as we intend to follow-up the patients and to control the effects of different treatment modes- the study is under way). MRI was performed within 3 days following US (in most patients both procedures took place on the same day). All MRI scans were assessed by the same MRI-qualified radiologist (M.Ż.) who was blinded to clinical details and US findings. Similarly to what was used in US, CET status on MRI was classified in a four-grade scale (i.e. high-grade tear, low-grade tear, suspected tear or no tear) and dichotomous scale (i.e. confirmed or unconfirmed tear).

### Statistical methods

Standard procedures were used for descriptive statistics. Sensitivity, specificity, positive predictive value (PPV), and negative predictive value (NPV) of US for detecting CET tear, using MRI as the reference, were calculated. The harmonic mean of recall and precision (F-score) was used to evaluate US accuracy. Agreement between US and MRI was measured using the weighted Cohen kappa coefficient (κ). According to Landis and Koch [7], the kappa values were interpreted as follows: 0.81–1.00 = very good agreement, 0.61–0.80 = good agreement, 0.41–0.60 = moderate agreement, 0.21–0.40 = fair agreement, 0–0.20 = poor agreement, and 0 = no agreement. The checklist that shows the completeness of reporting of diagnostic accuracy is presented in S2 File.

## Results

**Fig 1:**
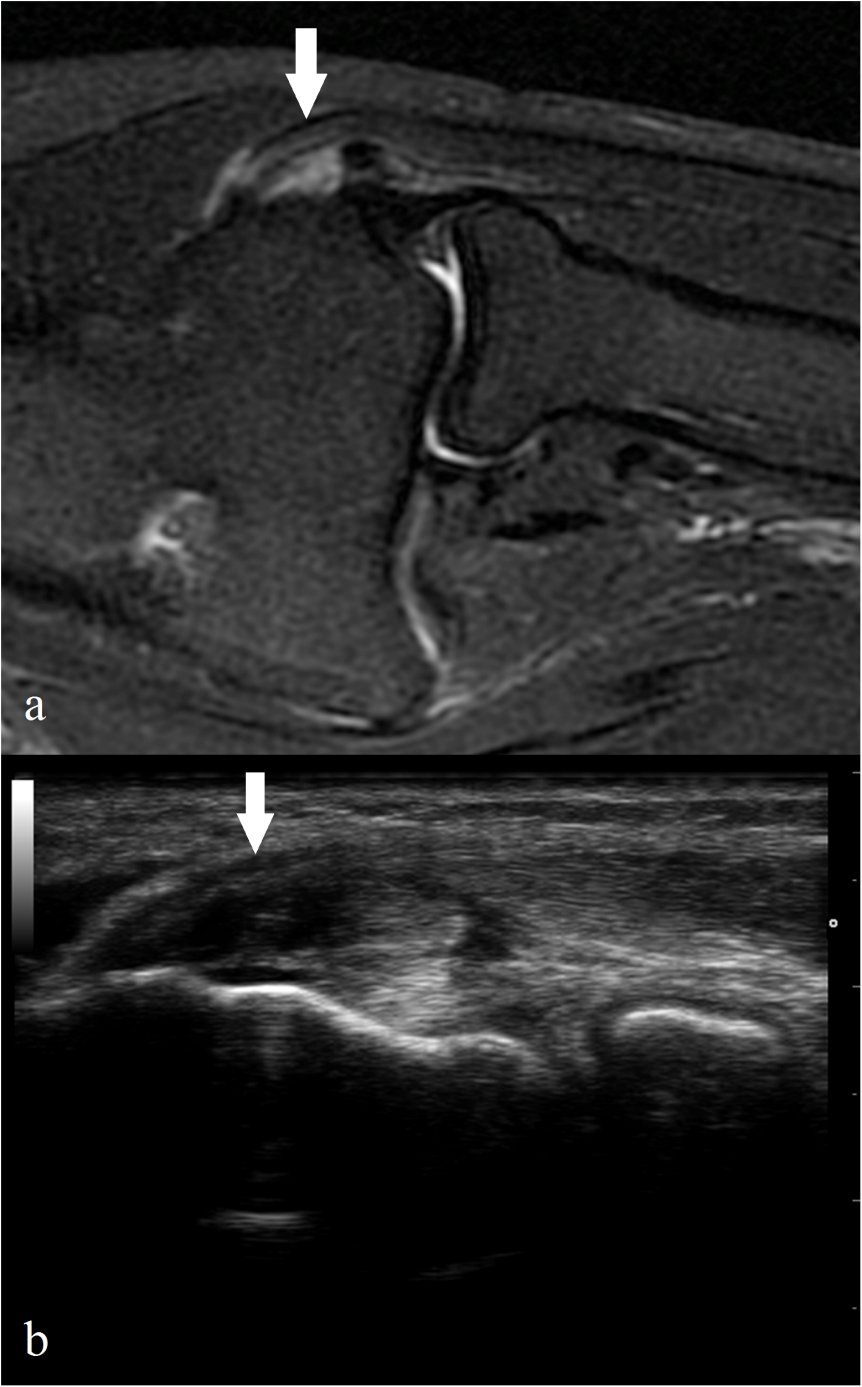
The images of high-grade CET tear (arrows) in MRI and US. Coronal fat-suppressed FSE T2-weighted image shows high-grade CET tear affecting more than 50% of the CET thickness (a). The corresponding US image of the CET tear in the same patient in longitudinal axis (b).

Demographic and clinical characteristics of the study group are shown in Table 1. The comparisons between MRI and US CET tear grading are presented in Table 2, Table 3 and S1 Table. On the basis of the dichotomous scale, US was in moderate agreement with MRI in detecting CET tear—κ = 0.49 (CI: 0.26–0.71). A similar agreement was found for the four-grade tear scale—κ = 0.49 (CI: 0.33–0.65). Using MRI as a reference, the sensitivity, specificity, and accuracy for the detection of CET tear by US were 64.52%, 85.19%, and 72.73%, respectively (Table 3). PPV and NPV of US as compared to MRI were 83.33% and 67.65%, respectively. The images of high-grade CET tear in MRI and US are presented in Fig 1.

**Table 1:** Demographic and clinical characteristics of the patients.

	Value
**Age, mean (range), years**	48 (27–71)
**Sex, male/female**	34/24
**Disease duration, mean (range), months**	15 (3–144)
**Physiotherapy, n(%)**	58 (100%)
**Corticosteroid injections, n(%)**	14 (24.1%)
**Shock-wave therapy, n(%)**	4 (6.9%)
**Nonsteroidal anti-inflammatory drugs > 2 weeks, n(%)**	3 (5.2%)

**Table 2:** Agreement between ultrasonography (US) and magnetic resonance imaging (MRI).

		MRI			
		high-grade CET tear (n)	low-grade CET tear (n)	Suspected CET tear (n)	no CET tear (n)
**US**	high-grade CET tear (n)	3	3	0	0
	low-grade CET tear (n)	4	10	2	2
	suspected CET tear (n)	0	9	8	4
	no CET tear (n)	0	2	3	8

CET- common extensor tendon; high-grade CET tear- a tear involving ≥50% of the CET thickness; low-grade CET tear- a tear involving <50% of the CET thickness; (n)–number of cases

**Table 3:** Sensitivity, specificity, positive predictive value (PPV) and negative predictive value (NPV) of ultrasonography (US) compared with magnetic resonance imaging (MRI) as the gold standard.

			MRI		
			Confirmed	Unconfirmed	
			CET tear (n)	CET tear (n)	
**US**	Confirmed CET tear (n)		20	4	PPV = 83.33%
	Unconfirmed CET tear (n)		11	23	NPV = 67.65%
			Sensitivity = 64.52%	Specificity = 85.19%	

CET- common extensor tendon; confirmed CET tear- evident CET tear on US/MRI; unconfirmed CET tear- no CET tear or suspected CET tear on US/MRI; (n)–number of cases

## Discussion

LE is a frequently encountered complaint in general practice with an incidence of 4–7/1000/year [8]. Imaging is not routinely indicated for LE diagnosis but is recommended in recalcitrant cases. Conservative treatment is efficient in most patients with LE and surgical interventions are reserved for those patients who remain symptomatic for 3 to 6 months despite treatment. However, Clarke et al. [6] suggested a lower threshold for surgery in patients with high-grade CET tears. Thus, identifying patients with high-grade CET tears seems to be important in predicting disease outcome and may be helpful in determining optimal treatment strategy. MRI can be regarded as the gold standard for LE diagnosing. However, taking in consideration the cost, availability, and several contraindications, MRI cannot be considered as a screening tool for CET tear detection in large cohorts of patients. US has many potential benefits over MRI. It is less costly and more accessible than MRI, and lacks any contraindications. A study by Levin et al. [9] showed that US has a relatively high sensitivity but low specificity in the detection of symptomatic LE and the authors suggested that US may be most useful for determining the extent of tendon damage in symptomatic patients.

The purpose of this study was to examine the reliability of US in detecting CET tear, using MRI as a reference standard. To our knowledge this is the first study comparing directly US to MRI in assessing CET tendon injury in chronic LE. Our findings showed good sensitivity (64.52%) and accuracy (72.73%), and very good specificity (85.19%) of US (versus MRI) in detecting CET tear. An important finding is that all patients with high-grade CET tear on US had confirmed CET tear on MRI. At the same time no patient with unconfirmed CET tear on US had high-grade CET tear on MRI. Thus, despite of moderate agreement between US and MRI in grading CET tear (κ = 0,49), high-grade tear in US can be considered as a reliable equivalent of confirmed tear. On the other hand, lack of evident tear in US virtually excludes the presence of high-grade CET tear.

Our results are in agreement with previous studies which showed that US is a reliable method to evaluate tendino-ligamentous structures of the lateral elbow region and the results of the US assessment are comparable to those of MRI [10–12]

We acknowledge several limitations of the study. First, it was a single-center study with both MRI’s and US’ evaluated by single observers without the assessment of inter- and intra-reader variability, thus the clinical usefulness of our results should be confirmed in a multi-center study. Second, we performed contrast MRI, which is not typical for elbow MRI in routine clinical practice. Contrast MR images were performed for the purpose of future evaluation, while CET tendon injury was assessed with the use of non-contrast MR images. However, it should not influence the reliability of the CET tendon injury assessment, as previous studies showed that administration of paramagnetic contrast agent in MR imaging of chronic epicondylitis does not provide additional information [13].

## Conclusions

Our study suggests that US is a valuable imaging modality that can be used as a screening tool to exclude high-grade CET tear in LE patients. Once a tear is evident on US, MRI should be considered to assess precisely the extent of tendon injury.

## Supporting information

S1 Table Common extensor tendon (CET) status in particular patients in magnetic resonance imaging (MRI) and ultrasonography (US). High-grade CET tear- a tear involving ≥50% of the CET thickness; low-grade CET tear- a tear involving <50% of the CET thickness.
(DOCX)

S1 File Standards for Reporting Diagnostic Accuracy (STARD) diagram. The diagram shows flow of participants through the study.
(JPG)

S2 File Standards for Reporting Diagnostic Accuracy (STARD) checklist. The checklist shows the completeness of reporting of diagnostic accuracy.
(DOCX)
