# Supplementary material for: Ultrasonography versus magnetic resonance imaging in detecting and grading common extensor tendon tear in chronic lateral epicondylitis
Source: PLoS One. 2017 Jul 27;12(7):e0181828. doi: 10.1371/journal.pone.0181828 (PMC5531525; doi:10.1371/journal.pone.0181828)
Supplement: S1 Table — High-grade CET tear- a tear involving ≥50% of the CET thickness; low-grade CET tear- a tear involving <50% of the CET thickness. (DOCX) [file pone.0181828.s001.docx]

**S1 Table. Common extensor tendon (CET) status in particular patients in magnetic resonance imaging (MRI) and ultrasonography (US).**

| **Patient No** |  | **CET status in MRI** |  | **CET status in US** |
| --- | --- | --- | --- | --- |
| **1** |  | low-grade tear |  | low-grade tear |
| **2** |  | suspected tear |  | suspected tear |
| **3** |  | no tear |  | no tear |
| **4** |  | low-grade tear |  | low-grade tear |
| **5** |  | high-grade tear |  | low-grade tear |
| **6** |  | suspected tear |  | low-grade tear |
| **7** |  | high-grade tear |  | high-grade tear |
| **8** |  | low-grade tear |  | low-grade tear |
| **9** |  | low-grade tear |  | low-grade tear |
| **10** |  | high-grade tear |  | high-grade tear |
| **11** |  | no tear |  | suspected tear |
| **12** |  | low-grade tear |  | low-grade tear |
| **13** |  | no tear |  | low-grade tear |
| **14** |  | no tear |  | suspected tear |
| **15** |  | suspected tear |  | no tear |
| **16** |  | low-grade tear |  | low-grade tear |
| **17** |  | no tear |  | no tear |
| **18** |  | low-grade tear |  | low-grade tear |
| **19** |  | low-grade tear |  | high-grade tear |
| **20** |  | high-grade tear |  | low-grade tear |
| **21** |  | suspected tear |  | suspected tear |
| **22** |  | low-grade tear |  | suspected tear |
| **23** |  | suspected tear |  | suspected tear |
| **24** |  | no tear |  | low-grade tear |
| **25** |  | high-grade tear |  | high-grade tear |
| **26** |  | no tear |  | no tear |
| **27** |  | suspected tear |  | suspected tear |
| **28** |  | no tear |  | no tear |
| **29** |  | suspected tear |  | suspected tear |
| **30** |  | suspected tear |  | suspected tear |
| **31** |  | low-grade tear |  | no tear |
| **32** |  | high-grade tear |  | low-grade tear |
| **33** |  | low-grade tear |  | suspected tear |
| **34** |  | low-grade tear |  | suspected tear |
| **35** |  | low-grade tear |  | suspected tear |
| **36** |  | low-grade tear |  | low-grade tear |
| **37** |  | low-grade tear |  | high-grade tear |
| **38** |  | low-grade tear |  | no tear |
| **39** |  | low-grade tear |  | low-grade tear |
| **40** |  | high-grade tear |  | low-grade tear |
| **41** |  | suspected tear |  | no tear |
| **42** |  | no tear |  | no tear |
| **43** |  | low-grade tear |  | suspected tear |
| **44** |  | no tear |  | no tear |
| **45** |  | low-grade tear |  | suspected tear |
| **46** |  | low-grade tear |  | high-grade tear |
| **47** |  | suspected tear |  | suspected tear |
| **48** |  | suspected tear |  | suspected tear |
| **49** |  | no tear |  | suspected tear |
| **50** |  | low-grade tear |  | low-grade tear |
| **51** |  | no tear |  | no tear |
| **52** |  | no tear |  | suspected tear |
| **53** |  | low-grade tear |  | suspected tear |
| **54** |  | suspected tear |  | low-grade tear |
| **55** |  | no tear |  | no tear |
| **56** |  | low-grade tear |  | suspected tear |
| **57** |  | suspected tear |  | no tear |
| **58** |  | low-grade tear |  | suspected tear |

High-grade CET tear- a tear involving ≥50% of the CET thickness; low-grade CET tear- a tear involving <50% of the CET thickness.
