# Supplementary figures and images for: Ultrasonography versus magnetic resonance imaging in detecting and grading common extensor tendon tear in chronic lateral epicondylitis
Source: PLoS One. 2017 Jul 27;12(7):e0181828. doi: 10.1371/journal.pone.0181828 (PMC5531525; doi:10.1371/journal.pone.0181828)

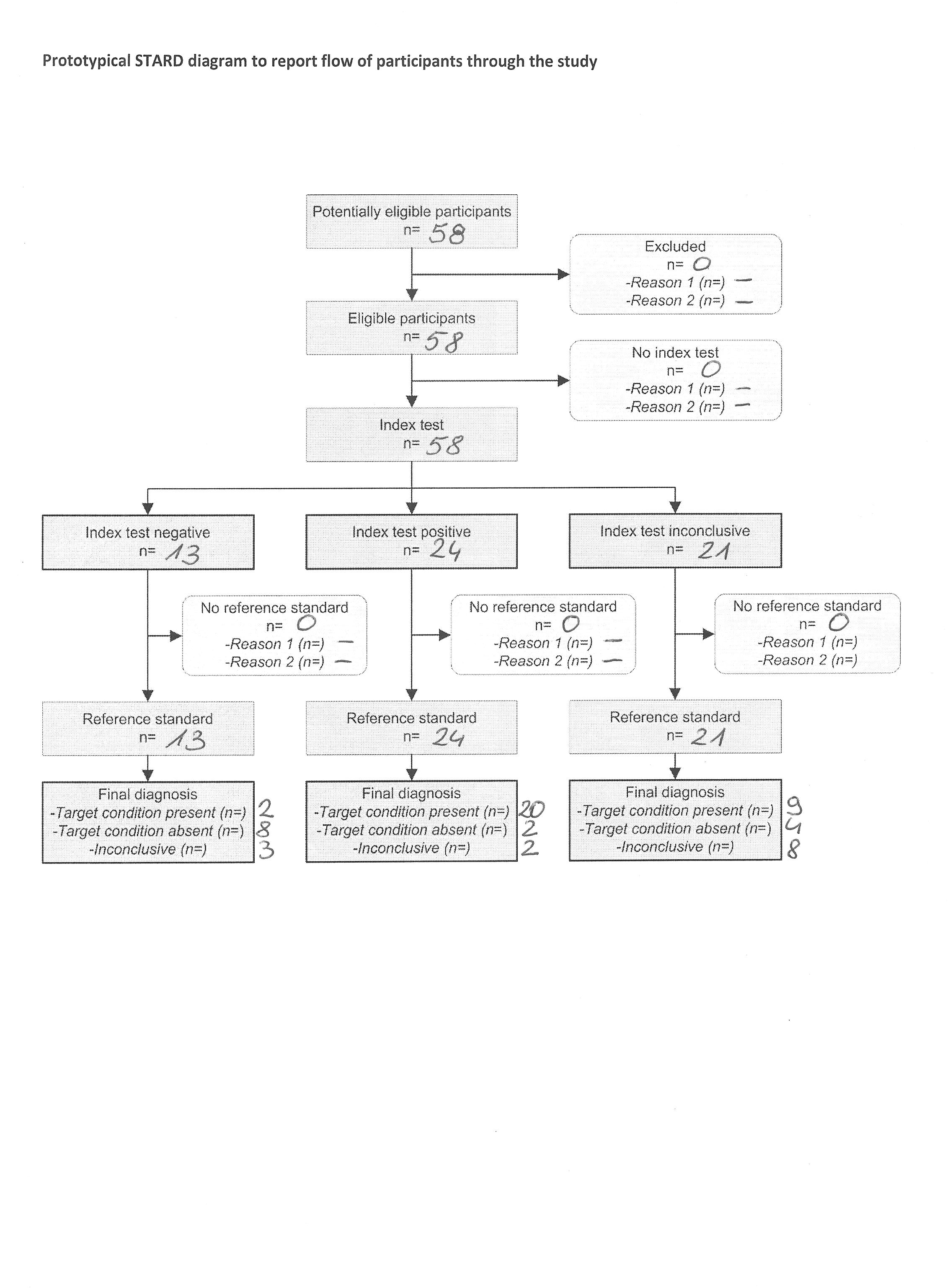

Supplement: S1 File — The diagram shows flow of participants through the study. (JPG) [file pone.0181828.s002.jpg]
